# Effective sensor properties and sensitivity considerations of a dynamic co-resonantly coupled cantilever sensor

**DOI:** 10.3762/bjnano.9.237

**Published:** 2018-09-25

**Authors:** Julia Körner

**Affiliations:** 1 University of Utah, 50 S. Central Campus Dr #2110, Salt Lake City, Utah, 84112, USA

**Keywords:** cantilever sensor, co-resonant coupling, effective sensor properties, sensor sensitivity

## Abstract

**Background:** Co-resonant coupling of a micro- and a nanocantilever can be introduced to significantly enhance the sensitivity of dynamic-mode cantilever sensors while maintaining the ease of detection. Experimentally, a low-stiffness nanocantilever is coupled to an easy to read out microcantilever and the eigenfrequencies of both beams are brought close to one another. This results in a strong interplay between both beams and, hence, any interaction applied at the nanocantilever alters the oscillatory state of the coupled system as a whole and can be detected at the microcantilever. The amplitude response curve of the microcantilever exhibits two resonance peaks and their response to an interaction applied to the sensor depends on the properties of the individual beams and the degree of frequency matching. Consequently, while an individual cantilever is characterized by its eigenfrequency, spring constant, effective mass and quality factor, the resonance peaks of the co-resonantly coupled system can be described by effective properties which are a mixture of both subsystem’s characteristics. These effective properties give insight into the amount of sensitivity of the nanocantilever that can be accessed and, consequently, into the sensitivity gain associated with the co-resonance. In order to design sensors based on the co-resonant principle and predict their behaviour it is crucial to derive a description for these effective sensor properties.

**Results:** By modeling the co-resonantly coupled system as a coupled harmonic oscillator and using electromechanical analogies, analytical expressions for the effective sensor properties have been derived and discussed. To illustrate the findings, numerical values for an exemplary system based on experimental sensor realizations have been employed. The results give insight into the complex interplay between the individual subsystem’s properties and the frequency matching, leading to a rather large parameter space for the co-resonant system’s effective properties. While the effective spring constant and effective mass mainly define the sensitivity of the coupled cantilever sensor, the effective quality factor primarily influences the detectability. Hence, a balance has to be found in optimizing both parameters in sensor design which becomes possible with the derived analytic expressions. Besides the description of effective sensor properties, it was studied how the thermal noise and, consequently, minimal detectable frequency shift for the co-resonantly coupled sensor represented by a coupled harmonic oscillator could be derived. Due to the complex nature of the coupled system’s transfer function and the required analysis, it is beyond the scope of this publication to present a full solution. Instead, a simplified approach to estimate the minimal detectable frequency shift for the co-resonant system based on the effective sensor properties is given.

**Conclusion:** By establishing a theoretical description for the effective sensor properties of a co-resonantly coupled system, the design of such systems is facilitated as sensor parameters can easily be predicted and adapted for a desired use case. It allows to study the potential sensitivity (gain) and detectability capabilities before sensor fabrication in a fast and easy way, even for large parameter spaces. So far, such an analysis of a co-resonantly coupled sensor was only possible with numerical methods and even then only with very limited capability to include and understand the complex interplay between all contributions. The outlined calculation steps regarding the noise considerations in a coupled harmonic oscillator system can provide the basis for a thorough study of that question. Furthermore, in a broader scope, the investigations presented within this work contribute towards extending and completing the already established theoretical basics of this novel co-resonant sensor concept and open up new ways of studying the coupled system’s behaviour.

## Introduction

Dynamic-mode cantilever sensors are used for many different applications which include the detection of smallest masses [1–2], in situ observation of the growth of biological films [3], detection of trace analytes in gases (”artificial nose”) [4] and the investigation of properties of novel (nano)materials by scanning probe methods or magnetometry [5–7]. In contrast to static-mode operation, where the static bending of cantilever sensors is used as a measurement signal, the dynamic mode is based on exciting the beam to vibrations and monitoring its amplitude, resonance frequency and phase shift. These properties can be altered either due to a change of the cantilever’s properties (spring constant, mass) or an external force gradient. The oscillation detection is usually realized by laser-optical methods such as interferometry or deflectometry [8].

In many cases, the shift of the cantilever’s resonance frequency ω_0_ is measured and, hence, the sensitivity of a cantilever sensor can be defined as the obtainable frequency shift with respect to an external interaction. This interaction can either be a force gradient represented by Δ*k* or a mass change Δ*m* (either point mass at the beam’s end or distributed mass) which alters the cantilever spring constant *k* and/or its effective mass *m*_eff_. The frequency shift Δω is then given by:

[1]
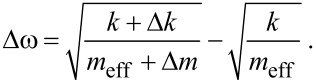


Assuming negligible mass change Δ*m* compared to the effective mass, the frequency shift becomes (see Supporting Information File 1 for details):

[2]
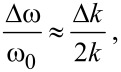


and for negligible change of the spring constant Δ*k* and a homogeneously distributed mass change:

[3]
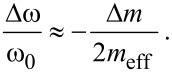


Please note that Equation 3 is derived from Equation 1 by a Taylor series expansion for small Δ*m* (see Supporting Information File 1 for details). From Equation 2 and Equation 3 it is evident that a small spring constant, small effective mass and high resonance frequency of the cantilever are favorable, especially for small interactions. Therefore, a common measure to increase the sensitivity of a cantilever sensor with regard to force gradients is the use of very soft beams which is typically achieved by reducing the cantilever dimensions, in particular the thickness. In this regard, attonewton force sensitivity has been demonstrated for very thin (≈100 nm) cantilever structures with a correspondingly low spring constant in the order of few µN/m [5]. Size reduction is also a favorable approach in terms of decreasing the effective cantilever mass. Another approach of reaching femtonewton force sensitivity has been demonstrated for optomechanical cantilever sensors by optimizing cantilever design for high quality factors (in the order of 10^6^). Consequently, cantilevers with rather high stiffness (kN/m) can be used, avoiding the snap-to-contact instability of very soft beams [9]. However, this experiment requires a highly specialized setup to drive the cantilever by optical means. In case of the typically used micromechanical cantilever sensors with piezo-actuator excitation, the low force sensitivity is achieved by reducing the beam’s size.

This reduction of the cantilever’s dimensions creates new challenges, not only for fabrication but also for oscillation detection [10]. Although sophisticated setups exist which allow the detection of the oscillatory state of a nanocantilever [11–12], these challenge hinders the widespread use of highly sensitive cantilevers with state-of-the-art equipment. Therefore, new concepts which access the high sensitivity of a nanocantilever but at the same time preserve the ease of oscillation detection need to be explored.

One approach is a recently introduced co-resonant measurement principle which combines the ease of detection at a microcantilever with the high sensitivity of a nanocantilever. The co-resonance is introduced by coupling these two beams and matching of their eigenfrequencies, i.e., they are brought close to one another, resulting in a strong interplay between both cantilevers. Thus, any interaction applied at the highly sensitive nanocantilever alters the oscillatory state of the coupled system as a whole and can be detected by measuring the coupled system’s amplitude response curve at the microcantilever [13–14]. Details about the basic underlying sensing principle as well as sensor fabrication with regard to coupling and eigenfrequency matching can for example be found in [13] and [15].

The sensor concept was tested experimentally in magnetic measurements. In cantilever magnetometry, individual Co_2_FeGa Heusler nanoparticles were studied with respect to their magnetic properties. This led to the first time observation of magnetic switching of these individual Heusler nanoparticles at room temperature and with a comparatively simple setup (laser-deflection detection) [16]. Other experiments in magnetic force microscopy showed a likewise increase in sensitivity [17–18].

These first proof-of-principle experiments and applications demonstrate the immense potential of the co-resonant sensor concept but they also indicate that a further study of the implications of the co-resonance is necessary.

As Equation 2 and Equation 3 indicate, the frequency shift response of an individual cantilever to an external interaction depends on the cantilever’s properties, i.e., its resonance frequency *f*, spring constant *k*, effective mass *m*_eff_ and also quality factor *Q* (with regard to detectability [19]).

While a single cantilever only exhibits one resonance peak for each of its oscillation modes, the coupled system’s amplitude response curve features two resonance peaks which show a differing frequency shift response to external influences on the system, depending on the degree of eigenfrequency matching. Furthermore, the frequency shift is always greater than that of the individual microcantilever and smaller than that of the individual nanocantilever. Consequently, the observation of the coupled system’s behavior leads to the conclusion that each resonance peak of the coupled system can be described by a set of effective sensor properties which are influenced by the characteristics of both individual beams and depend on the degree of eigenfrequency matching. These effective properties ultimately define the capabilities of the co-resonantly coupled system in terms of sensitivity and detectability. It is therefore crucial in view of sensor design and for evaluating sensor performance to derive ways of describing these effective properties and their dependence on the individual beam’s properties and the degree of eigenfrequency matching. Here, we present the derivation of simplified analytical formulas for the effective sensor properties of co-resonantly coupled sensor systems which will allow an accurate and fast way of determining prospective sensor performance. Furthermore, noise considerations within the coupled system and implications of the effective properties on the sensitivity and detectability of a co-resonantly coupled sensor are outlined.

In the following, first the sensitivity definition of a cantilever sensor will briefly be discussed and it will be evaluated how this can be used to estimate the sensitivity of a co-resonantly coupled cantilever system. This will allow to identify which effective properties of the coupled system are important in addition to the effective spring constant and effective mass which are already indicated by Equation 2 and Equation 3. In the next section, the modelling approach will be introduced. Then, the derivation of effective properties based on that model as well as the resulting expressions will be presented. These allow to estimate the potential performance and limitation of the system (e.g., sensitivity for a given task) before fabricating it and give new insights into the behaviour of co-resonantly coupled oscillating systems. Additionally, the treatment of thermal noise within the coupled system will be outlined.

## Sensitivity of a Cantilever Sensor

The sensitivity of a cantilever sensor is given by its minimal detectable frequency shift with respect to an external interaction. It is influenced by various noise contributions which are due to the cantilever itself (e.g., thermal noise, thermal frequency drift noise), the measurement principle (e.g., magnetic noise in case of magnetic measurements) and the excitation and detection setup (e.g., oscillator noise, detector noise) [8,20]. However, the lowest limit for a cantilever’s sensitivity is given by its thermal fluctuations leading to a thermally induced average frequency shift. This results in a minimal detectable frequency shift signal that is usually given in terms of a minimal detectable force gradient [5,8,21–22] or minimal detectable mass [23]. The following discussion will therefore be focused on the thermal noise limit and the minimal detectable frequency shift as a representation for sensitivity.

A cantilever can be described by a harmonic oscillator model with varying parameters for each of its eigenfrequencies [24] which is the basis used for the sensitivity considerations. The minimal detectable frequency shift ∂ω_th_ of an individual cantilever represented by a harmonic oscillator is given by [8]:

[4]
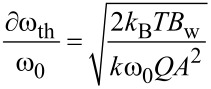


with the Boltzman constant *k*_B_, temperature *T*, measurement bandwidth *B*_w_ and the cantilever parameters spring constant *k*, quality factor *Q*, eigenfrequency ω_0_ and oscillation amplitude *A*.

Equation 4 is derived based on the equipartition theorem for a harmonic oscillator with one degree of freedom as outlined in [25] and [26]. It may also be applied to estimate the minimal detectable frequency shift and, hence, sensitivity of the coupled system based on the hypothesis that each of the resonance peaks of the coupled system can be represented by an effective harmonic oscillator with effective properties as outlined in [14]. Consequently, that requires the derivation of expressions for effective spring constant, resonance frequency and quality factor and a discussion about the oscillation amplitude of the co-resonant system.

## Modelling Approach

**Figure 1:**
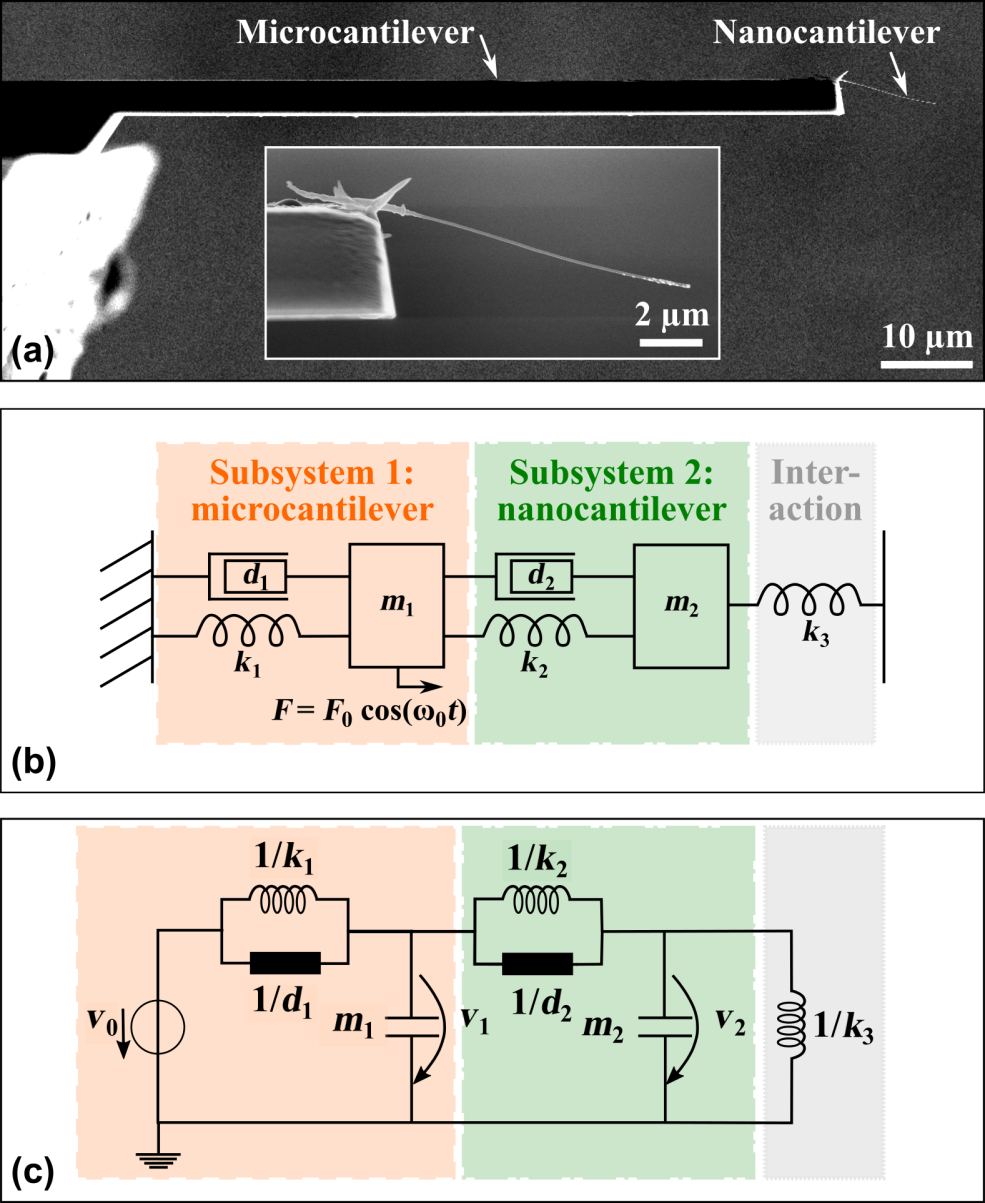
(a) Scanning electron microscopy image of a sensor realization consisting of a silicon microcantilever and a carbon nanotube nanocantilever. (b) Sensor’s representation by a coupled harmonic oscillator model and (c) corresponding electric circuit model. *m*_1,2_ denote the effective mass, *d*_1,2_ the damping and *k*_1,2_ the spring constant of each individual beam. The potential external interaction applied at the nanocantilever is modelled by an additional spring *k*_3_ (representing a force gradient) [14]. The system is excited via a periodic force *F* applied at the microcantilever.

The derivation of analytical expressions for the effective properties of the co-resonant system will be based on a modelling approach which has been discussed extensively in [13] and [14] and will therefore only briefly be outlined here. As mentioned above, the co-resonant cantilever system can be described as a coupled harmonic oscillator, consisting of a damping element *d*_1,2_, spring *k*_1,2_ and effective mass *m*_1,2_ for each subsystem. Please note that *m*_1,2_ still denotes the effective mass but the subscript *eff* was omitted to keep the descriptions short. The model furthermore allows to study external interactions on the coupled system and in Figure 1 a force gradient represented by an additional spring *k*_3_ is exemplarily considered.

By employing electromechanical analogies and the conventions force *F* ≡ current *I* and velocity *v* ≡ voltage *U*, the mechanical model can be transformed into an electric circuit [14]. The respective models are depicted in Figure 1 together with an experimental sensor representation. The circuit model gives the opportunity to utilize analytical (e.g., Laplace-space analysis) and simulation tools (e.g., Spice) to study the system’s behaviour.

All following considerations will be based on this model. In order to present some graphic representations of the analytical formulas derived below, the exemplary parameters given in Table 1 for a micro- and a nanocantilever will be used. They are based on sensor parameters which have been observed for experimental realizations of the co-resonantly coupled system such as depicted in Figure 1a.

**Table 1:** Numerical values for micro- (1) and nanocantilevers (2) based on experimental implementations of co-resonantly coupled sensors. Please note that the values are given for the initial state before frequency matching for the individual subsystems (columns 2 and 3). The two right-hand side columns summarize the values for both resonance peaks (*a* - left peak, *b* - right peak) of the coupled system in case of +2% eigenfrequency deviation Δ*f_e_* between micro- and nanocantilever (*f*_1_ = 200 kHz, *f*_2_ = 204 kHz) and have been calculated based on the model in Figure 1c.

	Individual subsystems		Coupled system, Δ*f_e_* = +2%	

Parameter	Micro (1)	Nano (2)	Left peak (a)	Right peak (b)

Frequency *f*	200.0 kHz	400.0 kHz	198.3 kHz	205.8 kHz
Spring constant *k*	1 N/m	0.001 N/m	0.0044 N/m	0.0013 N/m
Quality factor *Q*	10000	800	2670	1008
Effective mass *m*_eff_	6.33 × 10^−13^ kg	6.09 × 10^−16^ kg	2.83 × 10^−15^ kg	7.77 × 10^−16^ kg

**Figure 2:**
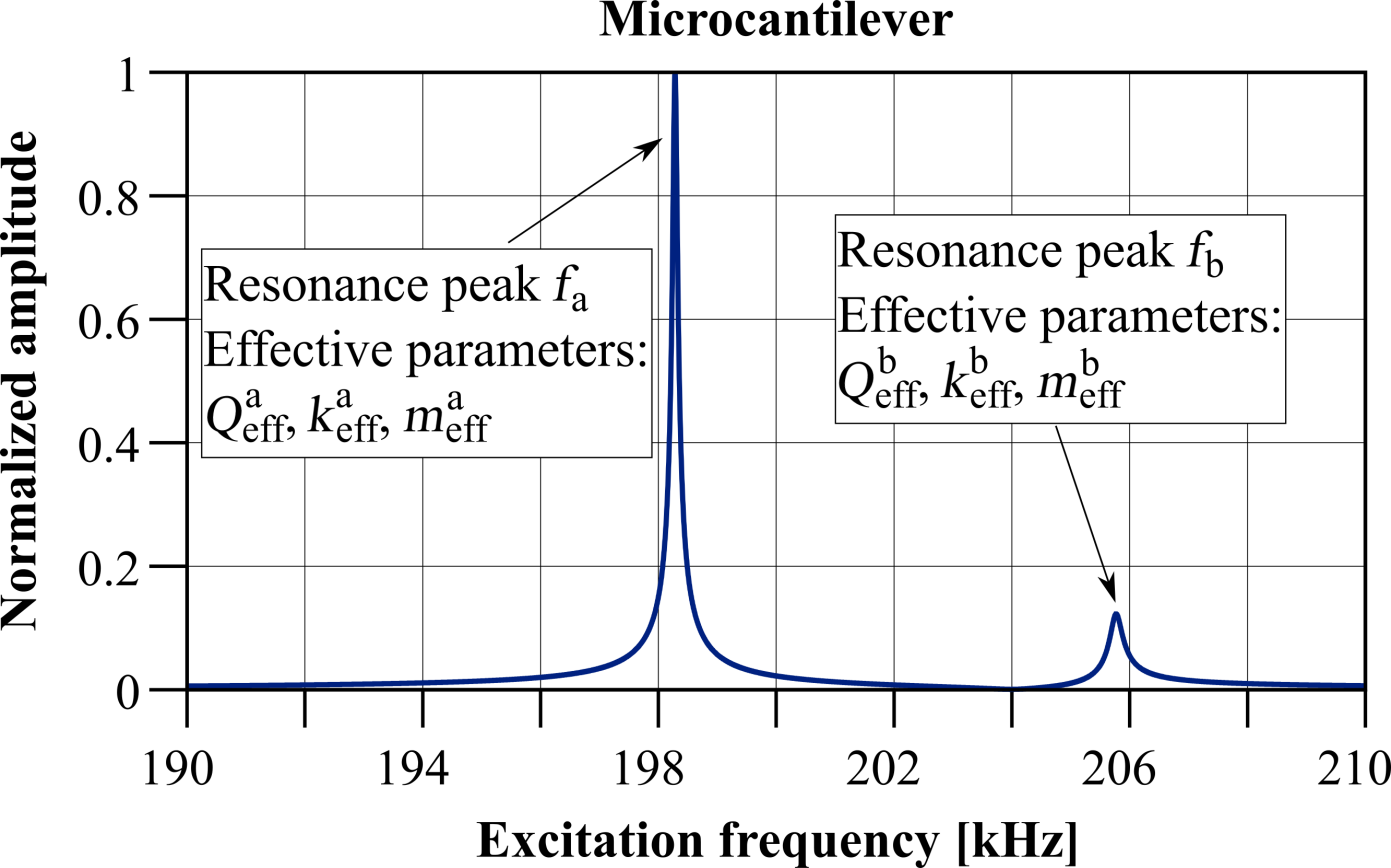
Calculated microcantilever amplitude response curve of the co-resonantly coupled system based on the values in Table 1 and for +2% eigenfrequency deviation between micro- and nanocantilever. The amplitude has been normalized to the maximum value of the curve.

Furthermore, please note that the following naming convention will be used to distinguish between the properties of the individual subsystems and those of the coupled system. For the individual subsystem, the indices 1 and 2 indicate micro- and nanocantilever, respectively. Indices *a* and *b* will be employed for the coupled system’s parameters, where *a* always refers to the left resonance peak (the one with the lower resonance frequency) and *b* to the right resonance peak (higher resonance frequency). To visualize these definitions, Figure 2 shows the amplitude response curve of the coupled system calculated for the microcantilever based on the circuit model from Figure 1c and for the values in Table 1 and +2% eigenfrequency deviation between micro- and nanocantilever. Additionally, most expressions will be derived in dependence on the angular frequency ω but in some cases this will be recalculated into the frequency *f* by ω = 2π*f*.

## Results and Discussion

### Derivation of effective sensor properties

The effective sensor properties which characterize each resonance peak are the resonance frequency *f*_a,b_, the effective spring constant 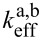 and effective quality factor 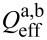. Furthermore, as described above, the relevant measured amplitude *A* of the coupled system has to be defined in order to employ the known sensitivity definition. This additionally warrants a discussion of thermal noise in the coupled system and an evaluation of how the thermally induced amplitude noise may be amplified due to the co-resonance and how that may affect the detection limit. In the following, the derivation of the effective properties based on the coupled harmonic oscillator model will be outlined and the implication on sensitivity and detectability will be discussed.

#### Resonance frequencies of a co-resonantly coupled system

To analytically derive the resonance frequencies for the co-resonantly coupled system, the circuit model in Figure 1c is considered. The resonance frequencies are found by determining the frequencies where the maxima of the amplitude response curve for subsystem 1 (microcantilever) *A*_1_(ω) = |*v*_1_/*v*_0_| and/or subsystem 2 (nanocantilever) *A*_2_(ω) = |*v*_2_/*v*_0_| occur. Previous investigations have shown that both amplitude response curves exhibit the same resonance frequencies, hence, only subsystem 1 will be considered in the following. Please note that the derivations are exactly the same if subsystem 2 is used [14]. Considering the damped coupled harmonic oscillator results in very complex expressions for the amplitude response curves (see [14]). Analytical calculation of the resonance frequencies would involve the derivative of the amplitude response curve to be zero which results in a sixth degree polynomial expression that can only be solved numerically.

Consequently, for an estimate of the resonance frequencies, we consider the model from Figure 1c without the damping elements *d*_1,2_. The validity of this assumption is supported by comparison of simulation results for the damped and undamped circuit model which show that the position of the resonance frequencies is only minimally influenced, even for high damping, i.e., low quality factors [14].

This can be understood by following the reasoning of [27]. In case of viscous damping, one has to distinguish between the angular natural frequency (eigenfrequency) ω_0_ and the angular frequency of damped vibration (resonance frequency) ω*_d_*. The former remains unchanged in case of damping as it only depends on the properties spring constant *k* and effective mass *m*_eff_ of the system itself, i.e., 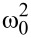 = *k*/*m*_eff_. The resonance frequency ω*_d_* is shifted compared to the eigenfrequency, depending on damping, hence ω*_d_* = 
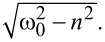
 Thereby, *n* denotes the ratio between damping coefficient *d* and effective mass *m*_eff_, i.e., *n* = *d*/2*m*_eff_. Employing this together with *d* = 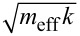/*Q* leads to:

[5]
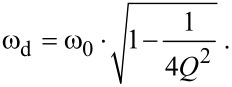


From Equation 5 it can be concluded that, even for low quality factors, the assumption from above gives a good approximation for the resonance frequencies of the coupled system.

In that case, the expressions for the amplitude response curves of micro- and nanocantilever read:

[6]
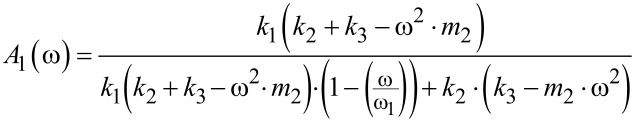


[7]
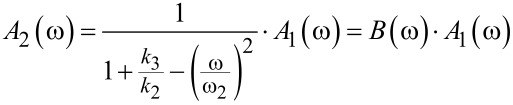


Here, *B*(ω) denotes an amplification factor between the amplitude of micro- and nanocantilever which is also frequency-dependent.

The corresponding resonance frequencies ω_a,b_ for left *a* and right peak *b*, respectively, are found by determining the poles of Equation 6, resulting in [13,28]:

[8]
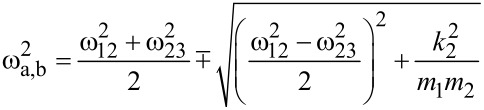


with the squared combined frequencies:

[9]
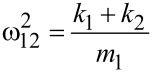


[10]
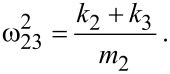


Please note that the radical term in Equation 8 is subtracted to calculate the left resonance peak *a* and the plus sign refers to resonance peak *b* with the higher resonance frequency. This is to ensure consistency with the definition of the resonance peaks given above.

By employing the relation ω^2^ = *k*/*m*, Equation 8 can be expressed by the eigenfrequencies ω_1,2_ of micro- and nanocantilever:

[11]
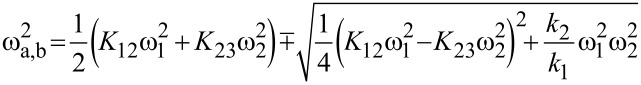


with

[12]
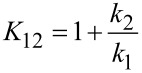


[13]
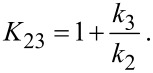


By assuming a constant eigenfrequency of subsystem 1, i.e., ω_1_ = const, and that only the eigenfrequency of subsystem 2 is varied, the coupled resonance frequencies can be derived in dependence on the degree of eigenfrequency matching Δω_eigen_ (see Supporting Information File 1 for details):

[14]
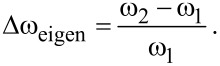


**Figure 3:**
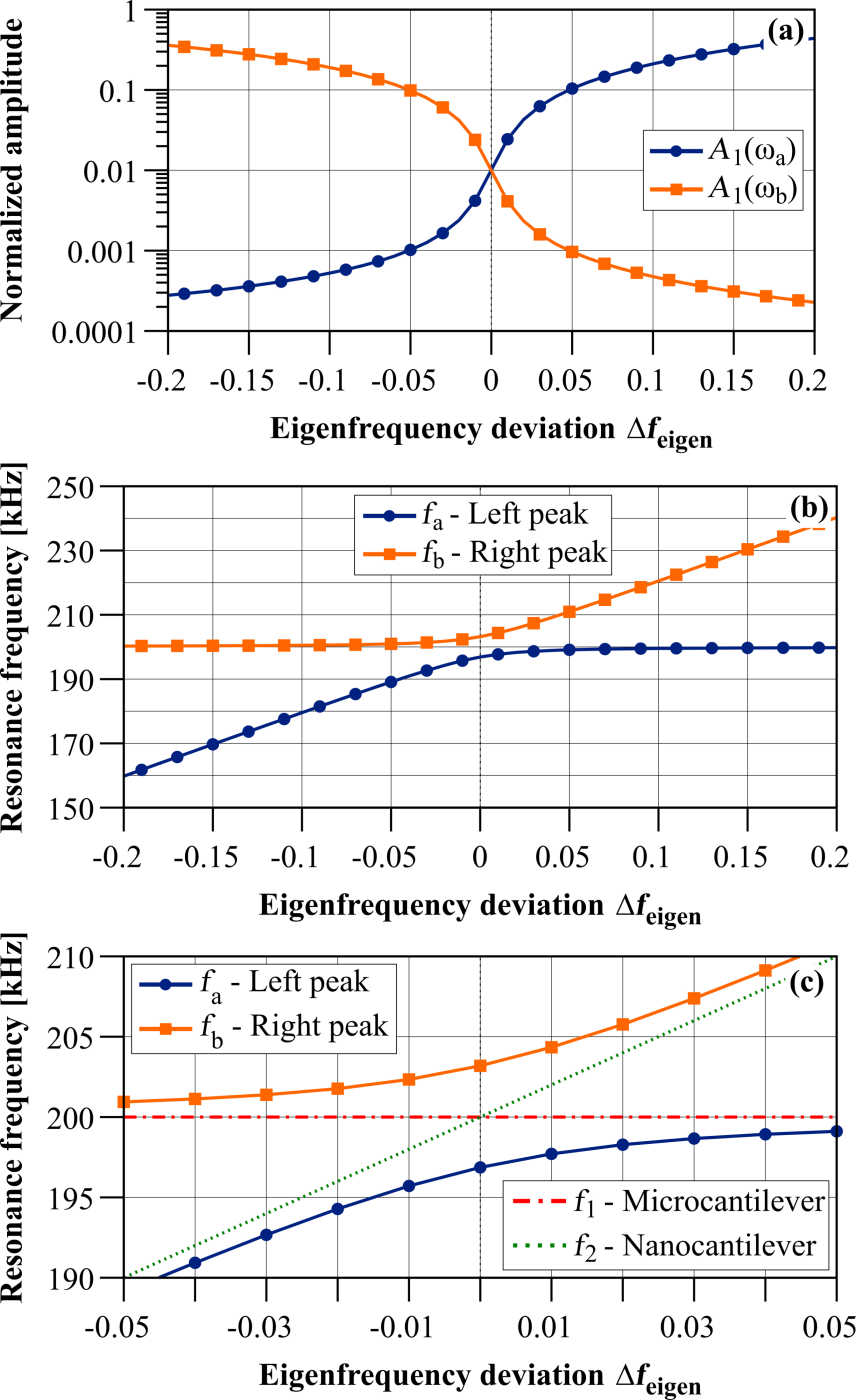
(a) Resonance amplitudes of both resonance peaks of the coupled system calculated for the microcantilever (index 1) and (b) resonance frequencies of both resonance peaks of the coupled system in dependence on the eigenfrequency deviation Δ*f*_eigen_ = Δω_eigen_/2π based on Equation 11 for the values given in Table 1. (c) Magnification of the resonance frequencies of both resonance peaks of the coupled system for small eigenfrequency deviation with added eigenfrequencies of micro- and nanocantilever to illustrate the effect of the co-resonance.

Please note that Δω_eigen_ is a dimensionless quantity which will be used to generate all following graphs. Figure 3 depicts the resonance frequencies for both resonance peaks of the coupled system in dependence on the eigenfrequency deviation Δ*f*_eigen_ = Δω_eigen_/2π based on Equation 11 as well as the amplitudes of the resonance peaks. In case of negative eigenfrequency deviation, i.e., *f*_2_ *< f*_1_, the lower left branch corresponds to the left resonance peak *a* which has a small amplitude and whose resonance frequency is changing because it corresponds to the nanocantilever. The upper left branch corresponds to the resonance peak *b* which has a high amplitude and is mainly corresponding to the microcantilever. For *f*_2_ ≈ *f*_1_ (see magnification in Figure 3c), the resonance frequencies for the coupled system clearly deviate from the eigenfrequencies of micro- and nanocantilever, which is also the region where the interplay between both beams is strongest. Hence, in that region, the effective properties of the coupled system’s resonance peaks will have a significant contribution of both individual beam’s properties. Please note that this is also the region where the so called ”avoided crossing” of the resonance frequencies is clearly visible which has already been described for coupled oscillating systems [29] and for the co-resonant approach in particular in [14]. In case of positive eigenfrequency deviation, i.e., *f*_2_ *> f*_1_, the upper right branch in Figure 3a corresponds to the left resonance peak *a* which now has the higher amplitude and is approaching the eigenfrequency of and mainly corresponding to the properties of the microcantilever. The lower right branch in Figure 3a corresponds to the right resonance peak *b* whose amplitude is decreasing with increasing eigenfrequency deviation and whose properties increasingly correspond to that of the nanocantilever. Please note that the discussion of the amplitudes is only valid if the amplitude response curve of the microcantilever is studied. If the nanocantilever would be considered, the amplitude of the smaller resonance peak would be significantly increased but the conclusions regarding coupled resonance frequencies and effective properties are the same as for the microcantilever’s amplitude response curve.

#### Effective spring constant

**Figure 4:**
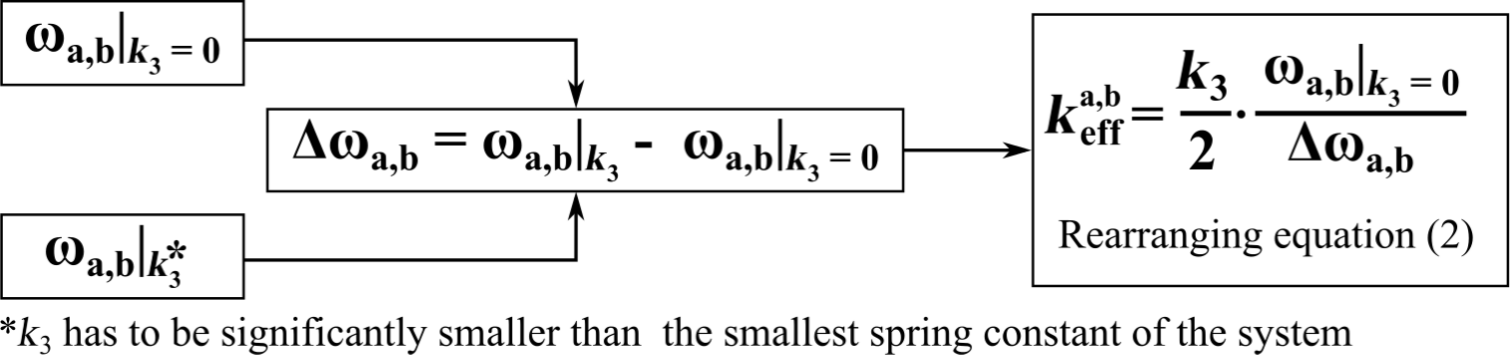
This diagram represents the necessary calculation steps to determine the effective spring constant 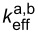 for both resonance peaks of the coupled system.

Based on the expressions for the resonance frequencies of the coupled system, the effective spring constants for both resonance peaks 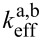 can easily be derived by using Equation 2. Figure 4 gives an overview of the calculation steps.

Equation 2 is originally given for a single cantilever (harmonic oscillator) but can be employed for a coupled harmonic oscillator by using the resonance frequencies of the coupled system and the effective spring constants 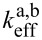 instead of *k*. The first step is the calculation of both resonance frequencies ω_a,b_ of the coupled system, with and without an interaction Δ*k* = *k*_3_. As shown in [15], it is crucial to choose *k*_3_ to be much smaller (at least two orders of magnitude) than the smallest spring constant of the coupled system. Otherwise, the effective spring constant will strongly depend on *k*_3_ [30]. Please note that this is the case for any cantilever sensor and not a feature of the co-resonantly coupled system.

Rearranging Equation 2 results in:

[15]
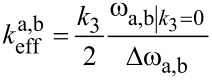


with

[16]
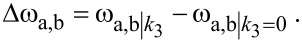


**Figure 5:**
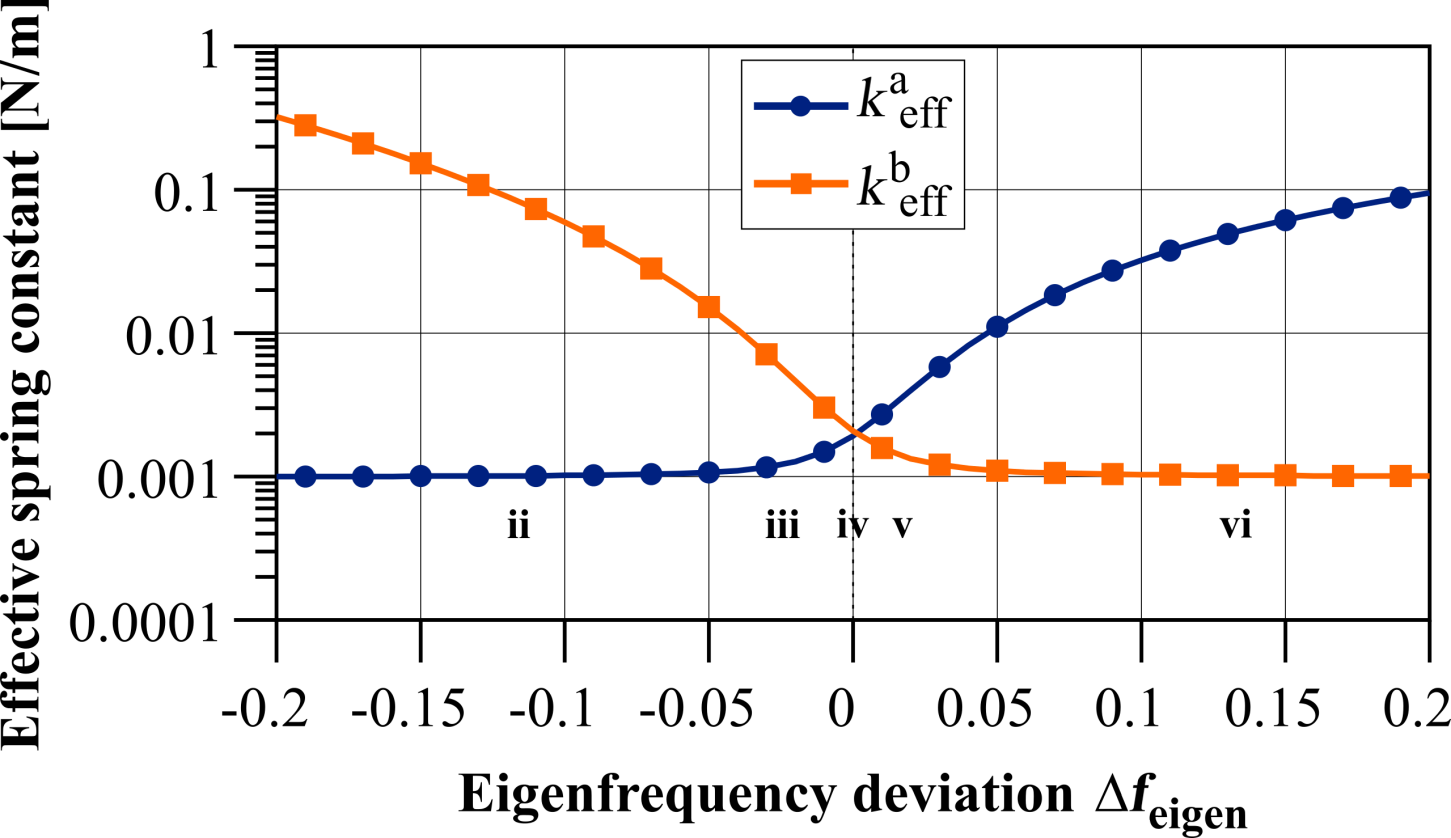
Effective spring constants for both resonance peaks of the coupled system in dependence on the eigenfrequency deviation Δ*f*_eigen_ = Δω_eigen_/2π based on Equation 15 for the values given in Table 1. Different sections can be identified which are summarized in Table 2 and sections ii to vi are depicted in the graph.

By substituting ω_2_ as given in Equation 14, the effective spring constants for the co-resonantly coupled system can be given as a function of the degree of eigenfrequency matching (see Supporting Information File 1 for details). Figure 5 depicts the effective spring constants for left (a) and right (b) resonance peak of the coupled system based on Equation 15 for the values given in Table 1.

In Figure 5, different sections can be identified which are summarized in Table 2. These sections are of general nature and not specific for the exemplary values given in Table 1. However, it is important to note that the width of the sections depends on the properties of both subsystems and that the slope of the transition region, i.e., sections ii, iii, v and vi is dependent on the ratio of the individual beam’s spring constants. The greater the difference between the individual spring constants, the steeper the slope.

**Table 2:** Overview of amplitude and effective spring constants of both resonance peaks of the coupled system in dependence on the eigenfrequency deviation. Seven general sections of the curve depicted in Figure 5 can be identified. Please note that the result for section iv has already been described by T. Mühl [31] and in [8].

Section	Frequency relation	Amplitude relation	Left peak 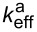	Right peak 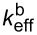

i	*f*_2_ *<< f*_1_	*A*_a_ *<< A*_b_	= *k*_2_	= *k*_1_
ii	*f*_2_ *< f*_1_	*A*_a_ *< A*_b_	≈ *k*_2_	2*k*_2_ *<* 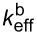 *< k*_1_
iii	*f*_2_ ≈ *f*_1_	*A*_a_ *< A*_b_	*k*_2_ *<* 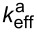 *<* 2*k*_2_	2*k*_2_ *<* 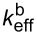 *< k*_1_
iv	*f*_2_ = *f*_1_	*A*_a_ ≈ *A*_b_	= 2 · *k*_2_	= 2 · *k*_2_
v	*f*_2_ ≈ *f*_1_	*A*_a_ *> A*_b_	2*k*_2_ *<* 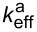 *< k*_1_	*k*_2_ *<* 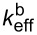 *<* 2*k*_2_
vi	*f*_2_ *> f*_1_	*A*_a_ *> A_b_*	2*k*_2_ *<* 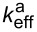 *< k*_1_	≈ *k*_2_
vii	*f*_2_ *>> f*_1_	*A*_a_ *>> A*_b_	= *k*_1_	= *k*_2_

Furthermore, a slight asymmetry can be found in the curve which is due to the very different properties of the individual subsystems, and, consequently, leads to an asymmetric energy distribution in the coupled system which is reflected in the effective sensor properties. That is not only the case for the effective spring constants but also for the effective quality factor as well as amplitude considerations.

Although the expressions without damping have been used, a comparison of effective spring constants obtained by Spice simulations of the damped co-resonantly coupled system and the undamped analytical calculations based on Equation 15 shows a good agreement, even for rather large damping, i.e., low quality factors. For the exemplary values given in Table 1 and with quality factors below 100 for both subsystems, the deviation between the effective spring constants for undamped and damped case was less than 5%. Experimentally, the spring constant of a cantilever can be determined by various approaches such as thermal noise, Sader, Cleveland (added mass) methods [32–33] but they all have an uncertainty of at least 10% [33]. These comparisons indicates that the simplified expressions based on the undamped case give a good estimate for the effective spring constants of the coupled system.

#### Effective quality factor

The quality factor can either be defined as the ratio of total energy to dissipated energy per oscillation period [34] or as the bandwidth of the resonance curve. In the latter case, the bandwidth is given by the difference of the two frequencies at which the amplitude has been decreased to 1/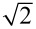 times the resonance amplitude [35]. Both definitions are equivalent for sufficiently low damping [36]. However, for the following derivation of the effective quality factor 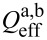 for both resonance peaks *a*, *b* of the co-resonantly coupled system, the definition based on energy dissipation will be used, hence:

[17]
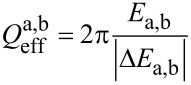


with *E*_a,b_ denoting the total energy stored in the system and Δ*E*_a,b_ the dissipated energy per oscillation period. Please note that the absolute value for the dissipated energy (often considered with a negative sign) is used as only the relation between total and dissipated energy is relevant for the quality factor.

Based on the coupled harmonic oscillator representation, the total energy of the system can be approximated by the potential energy given by [37]:

[18]
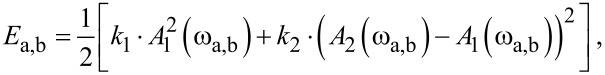


depending on the spring constants *k*_1,2_ and the amplitudes *A*_1,2_(ω_a,b_) at the resonance frequencies ω_a,b_.

The dissipated energy per oscillation cycle is defined by the damping coefficient *d*_1,2_ which contains all intrinsic and extrinsic damping contributions, and the oscillation speed *v*_1,2_(*t*):

[19]
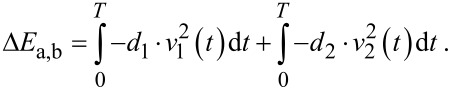


Assuming a deflection of *u*_1,2_(*t*) = *A*_1,2_·cos(ω*t*), the velocity of each oscillator is given by:

[20]
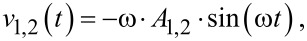


and hence, the dissipated energy is:

[21]
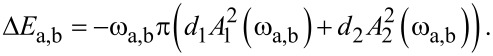


Employing *d*_1,2_ = 
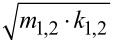
/*Q*_1,2_, *m*_1,2_ = *k*_1,2_/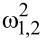 and the amplitude relation *A*_2_(ω_a,b_) = *B*_a,b_·*A*_1_(ω_a,b_) results in an effective quality factor for each resonance peak:

[22]
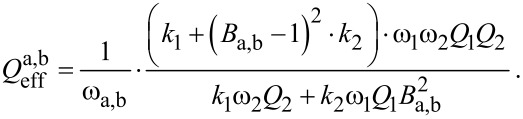


At this point it is necessary to discuss the factor *B*_a,b_ in more detail. It relates the amplitudes *A*_1_ and *A*_2_ of both subsystems as discussed above and for a model with integrated damping would be a complicated expression, resulting in a complex formula for the effective quality factor. To give a simplified estimate for the effective quality factor, the undamped relation is assumed which has been shown to be a reasonably good estimate. Therefore, *B*_a,b_ can be derived from Equation 7 for *k*_3_ = 0 and at the two resonance frequencies ω_a,b_:

[23]
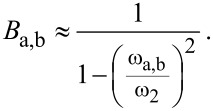


Equation 22 can also be expressed as a function of the eigenfrequency deviation Δω_eigen_ by substituting ω_2_ with the expression given in Equation 14, leading to:

[24]
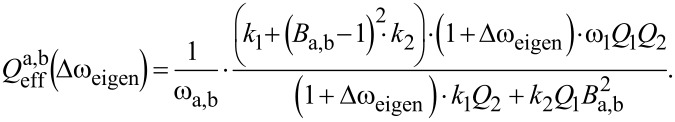


**Figure 6:**
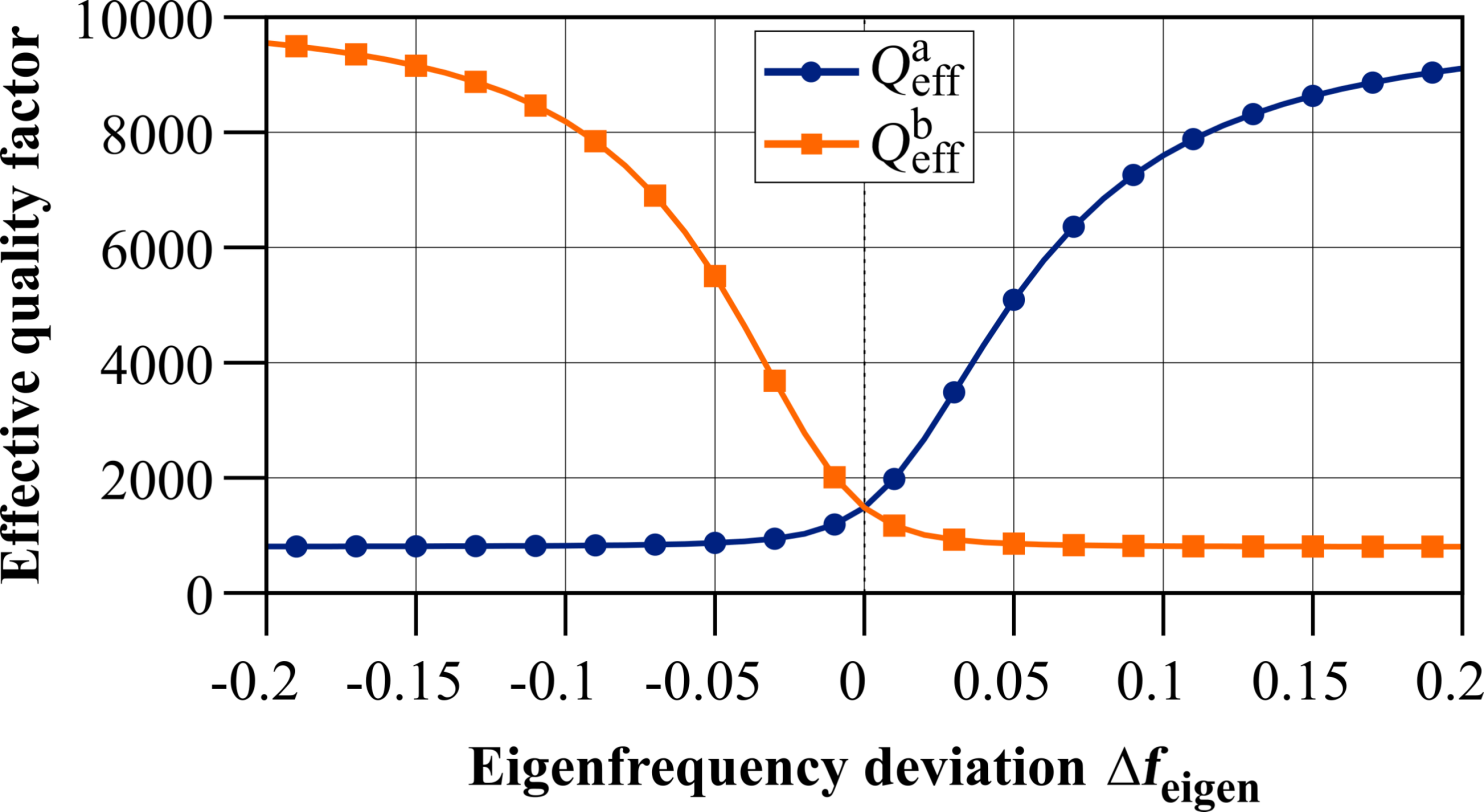
Effective quality factor for both resonance peaks of the coupled system in dependence on the eigenfrequency deviation Δ*f*_eigen_ = Δω_eigen_/2π based on Equation 24 for the values given in Table 1.

Figure 6 depicts the effective quality factors for both resonance peaks of the coupled system based on Equation 24 for the values given in Table 1. It shows a similar behaviour as the effective spring constants, leading to comparable sections as identified in Table 2.

In order to better understand the behaviour of the system in terms of the effective quality factor, it is instructive to make some assumptions. Two subsystems with the same quality factor, i.e., *Q*_1_ = *Q*_2_ = *Q*, are considered. This results in the following expression for the effective quality factor 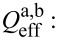


[25]
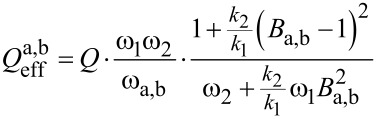


with

[26]
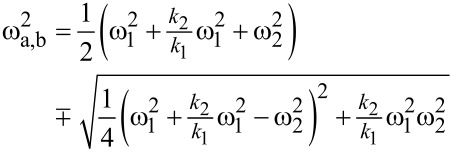


[27]
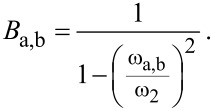


**Figure 7:**
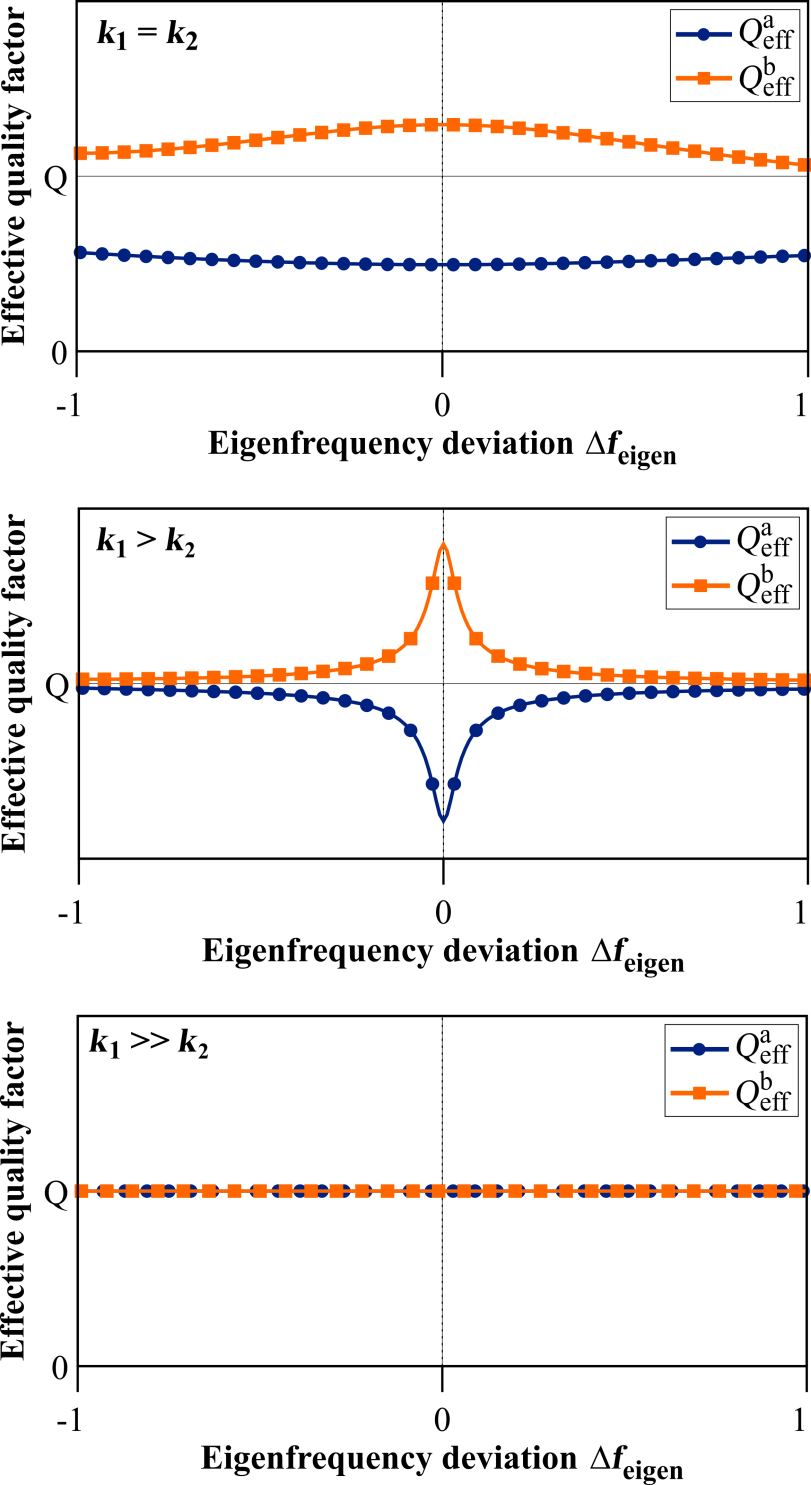
Qualitative behaviour of the effective quality factor for both resonance peaks of the coupled system in dependence on the eigenfrequency deviation Δ*f*_eigen_ = Δω_eigen_/2π for the special case of *Q*_1_ = *Q*_2_ = *Q* and varying ratio of the effective spring constants (see Equation 25).

All expressions in that case show a dependence on the ratio of the individual subsystem’s spring constants *k*_2_/*k*_1_ and Figure 7 illustrates the general behaviour of the effective quality factor of the co-resonantly coupled system’s resonance peaks for varying ratios of *k*_1_ and *k*_2_ and within an eigenfrequency deviation range of ±100%.

The case *k*_2_/*k*_1_ = 1 results in two different effective quality factors, one of which is smaller than *Q* and the other one is greater than *Q*:

[28]
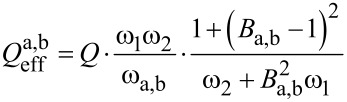


[29]
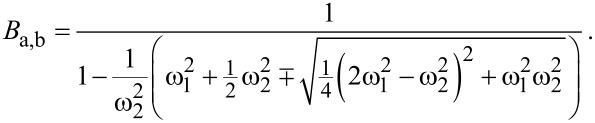


For *k*_2_/*k*_1_ *<* 1 the clearly separated lines from *k*_2_/*k*_1_ = 1 start to approach each other for greater frequency deviation but have a minimum/maximum respectively around zero eigenfrequency deviation. These extrema vanish for *k*_2_/*k*_1_ *<<* 1 resulting in the two effective quality factors being identical and equal to *Q*. This particular result has also been described by T. Mühl [31]. For the reversed case of *k*_2_/*k*_1_ *>* 1 the same behaviour as for *k*_2_/*k*_1_ = 1 is found but with increasing spacing between the two almost parallel lines when the difference between the two spring constants increases.

In general, i.e., without any assumptions for the individual subsystem’s properties, the slope of the curves for the effective quality factor depends on the ratio of the spring constants as well as the quality factors of the individual subsystems and different curve shapes can be found by varying the relations between all these parameters.

Furthermore, the effective quality factors calculated by the derived analytical expression from Equation 22 have been compared to values obtained by numerical circuit simulations of the coupled system with the software LTSpice. Thereby, a two step approach was used where first the amplitude response curve of the coupled system was simulated for varying properties of the subsystems and degree of frequency matching. In a second step, the quality factor of each resonance peak was determined by the definition based on the bandwidth. A wide range of parameters was simulated, for both, quality factors and spring constants of the subsystems and in all cases, a very good agreement was found between the analytical solution and the simulation.

Although the comparison between an analytical formula and simulations is somewhat limited by the parameter space covered in the simulation, the results strongly indicate that the derived analytical expression gives a very good estimate for the effective quality factors of a co-resonantly coupled system. Another conclusion drawn from the above discussion is that a more thorough study of the effective quality factor of co-resonantly coupled systems based on the obtained analytical expression will be necessary, especially with regard to tuning parameters for a sensor application. Here, only the case of *Q*_1_ = *Q*_2_ was discussed in detail and other relations will lead to a different behaviour compared to what is described above for this specific case. However, this is beyond the scope of this publication which aims at giving the basic relations for describing the coupled system’s effective properties.

### Measured amplitude and thermal noise

Equation 4 contains another parameter which is the oscillation amplitude *A* of the cantilever. The amplitude response curve of the coupled system is still measured at the microcantilever as in the case of an individual cantilever sensor. Therefore, no ”effective” value is required and the actual oscillation amplitude of the microcantilever can be used for the coupled system.

At this point, all effective properties of the coupled system have been derived and can be used to estimate the minimal detectable frequency shift for each resonance peak of the co-resonantly coupled system based on Equation 4. The important point which needs to be stressed here is that this approach is only valid under the assumption that each resonance peak of the co-resonant system can be modelled as an effective harmonic oscillator with effective properties.

While this is a reasonable assumption as shown in [14], the sensitivity discussion inevitably leads to the question of how the co-resonance is actually affecting thermal noise distribution in the coupled system and how that may affect the minimal detectable frequency shift. Finding an answer to that requires a derivation based on the equipartition theorem and transfer function for the coupled harmonic oscillator in a similar way as it is outlined in [25] for the individual harmonic oscillator. However, for the coupled system it has to be noted that while the complete system has to be considered in the derivation, any oscillation detection takes place at the microcantilever and therefore its amplitude is the quantity of interest.

The equipartition theorem states that, in thermal equilibrium, each independent quadratic term in the system’s total energy (i.e., each degree of freedom) equals a mean value of thermal energy 1/2*k*_B_*T* [26]. The coupled harmonic oscillator constitutes a two-degree of freedom system, hence the equipartition theorem in that case is:

[30]
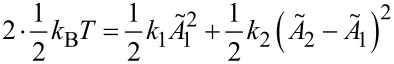


with the mean square displacement noise amplitudes 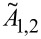 and spring constants *k*_1,2_ of subsystem 1 and 2. Rearranging Equation 30 and substituting 
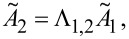
 the mean square thermal noise amplitude of the microcantilever becomes:

[31]
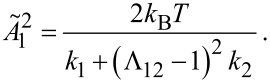


Please note that Λ_12_ is only equal to the previously used amplitude amplification factor *B*_a,b_ between micro- and nanocantilever in case of neglected damping. If the complete coupled harmonic oscillator model including damping is considered, the amplification factor becomes more complicated and a derivation thereof can be found in [14].

Following the reasoning of [25], the next steps involve the calculation of the white thermal noise density and the mean square displacement noise by integrating over the coupled system’s transfer function *G*(*f*) = *A*_1_/*A*_0_. By using the electric circuit model from Figure 1, *G*(*f*) = *v*_1_/*v*_0_ which can be found in [14]. The ansatz to be used for the noise calculation is:

[32]
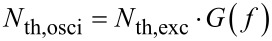


with the thermal oscillator noise density *N*_th,osci_ and the excitation white thermal noise density *N*_th,exc_. This represents the filtering of the excitation white thermal noise due to the resonance characteristics of the coupled harmonic oscillator system.

Integration of the squared thermal oscillator noise density from Equation 32 over the complete frequency range [0,∞] and relating that to the expression for the mean square amplitude noise obtained from the equipartition theorem (Equation 31 for the coupled harmonic oscillator) allows to derive the excitation white thermal noise density according to:

[33]
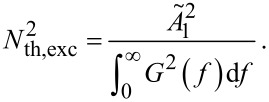


However, this involves solving the integral for the squared transfer function *G*^2^(*f*). While an antiderivative exists for the single harmonic oscillator, no solution is available for the coupled harmonic oscillator. Hence, numerical treatment is required and, based on this, it has to be studied if it is possible at all to find an approximation for an antiderivative for the given boundary conditions in the coupled case.

Integrating the oscillator noise over the relevant measurement bandwidth from *f*_low_ to *f*_high_ finally gives the mean square displacement noise according to

[34]
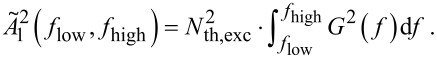


In case of a dynamic-mode cantilever which is excited at or close to its resonance frequency *f*_0_, the transfer function becomes *G*^2^(*f*_0_) = *Q*^2^ for a single harmonic oscillator. Again there is no solution for the co-resonantly coupled harmonic oscillator available yet.

The final derivation of the minimal detectable frequency shift is based on the ansatz:

[35]
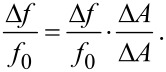


For a sensor working point close to the resonance frequency, the inverse slope of the amplitude response curve can be approximated by Δ*A*/Δ*f*_0_ ≈ *f*_0_/*QA* for a single harmonic oscillator [25]. It has to be studied in more detail but it is likely that this approximation still holds for the coupled harmonic oscillator since the resonance peaks in that case are measured in the same way as for the single harmonic oscillator. However, in this case *Q* would have to be replaced by the effective quality factor for the resonance peak measured and *A* = *A*_1_ since the amplitude of subsystem 1 (microcantilever) is used. Furthermore Δ*A* = 
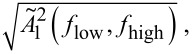
 and hence:

[36]
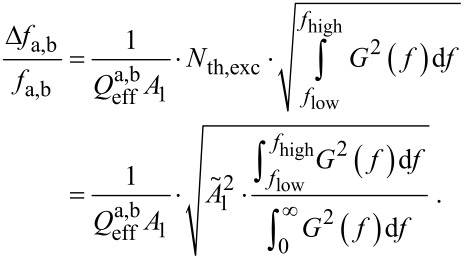


As Equation 36 and the above considerations show, two main questions have to be studied in order to derive an expression for the minimal detectable frequency shift of a co-resonantly coupled cantilever sensor represented by a damped coupled harmonic oscillator:

1. Derivation of an antiderivative or approximation formula thereof for the coupled system’s squared transfer function *G*^2^(*f*), including incorporation of the relevant boundary conditions
2. Approximation of inverse slope of the amplitude response curve close to the resonance frequency for each resonance peak

By solving these questions and following the steps outlined above, a complete analytical solution for the minimal detectable frequency shift and, consequently, the sensitivity of a co-resonantly coupled cantilever sensor may be derived. However, due to the co-resonantly coupled system constituting a two-degree of freedom system with a complex transfer function, the actual solution goes far beyond the scope of this publication which aims at providing simplified expressions for relevant sensor properties. The sensitivity calculation of a system represented by a coupled harmonic oscillator will therefore have to be part of future work as it will potentially involve numerical solutions and elaborate approximations and assumptions. Nonetheless, the steps outlined here can provide the basis for such an investigation.

### Implications on sensor sensitivity and detectability

Until a solution for the above open questions is found, the sensitivity based on the minimal detectable frequency shift of the co-resonantly coupled system can be estimated by using Equation 4 for each resonance peak based on the derived expressions for effective sensor properties. That furthermore allows to analyze the sensitivity gain induced by the co-resonant coupling in comparison to an individual cantilever sensor. For the exemplary values given in Table 1, the minimal detectable frequency shift can be calculated and consequently the minimal detectable force gradient and mass load for both individual subsystems as well as the resonance peaks of the coupled systems, in that case for +2% eigenfrequency deviation. For the calculations, Equation 2 and Equation 3 have been used with Δ*f*/*f*_0_ being substituted by the expression in Equation 4. The results are listed in Table 3 and illustrate how the co-resonant concept allows to access the high sensitivity of the nanocantilever.

**Table 3:** Minimal detectable frequency shift and related force gradient and mass load for individual micro- and nanocantilever and both resonance peaks of the coupled system represented by effective properties. For the calculation, Equation 4 has been used, assuming measurement bandwidth *B*_w_ = 1 Hz and room temperature *T* = 293 K.

	Individual subsystems		Coupled system, Δ*f*_e_ = +2%	

Parameter	Micro (1)	Nano (2)	Left peak (a)	Right peak (b)

Amplitude *A*	100 nm	1000 nm	100 nm	100 nm
Freq. shift Δ*f*_therm_	1.6 mHz	18.1 mHz	46.6 mHz	142.2 mHz
Min. force gradient *k*_min_	1.6 × 10^−8^ N/m	1.8 × 10^−10^ N/m	2.1 × 10^−9^ N/m	1.8 × 10^−9^ N/m
Min. mass *m*_min_	1.0 × 10^−20^ kg	1.1 × 10^−22^ kg	1.3 × 10^−21^ kg	1.1 × 10^−21^ kg

Please note that the estimates presented here are very conservative, as for example a rather high stiffness of the nanocantilever and room temperature were assumed. A decrease in stiffness, especially of the nanocantilever, and low temperatures will lead to much more gain in sensitivity. With the derived expressions within this work, a fast and easy way to estimate the potential sensitivity is given.

However, the co-resonantly coupled system’s frequency shift response to an external interaction is only one aspect. The other equally important aspect is the detectability, i.e., how well the oscillatory state of a dynamic-mode cantilever sensor can be detected. That is limited by the signal-to-noise ratio (SNR) for frequency, amplitude and phase measurements. It mainly depends on the cantilever’s quality factor which directly influences the phase noise and resolution of the resonance peak [19]. With decreasing cantilever dimensions, the quality factor usually decreases, hence, detectability deteriorates [38]. As the considerations for the effective quality factor of the co-resonantly coupled system show, this effect is, at least partly, counteracted by the co-resonance. While the high sensitivity of the nanocantilever is accessible, the usually higher quality factor of the microcantilever becomes beneficial for detectability. Hence, if always the resonance peak with the higher amplitude is measured, the effective quality factor will never be below approximately twice the smallest individual quality factor of the system. Furthermore, as the graphs for effective quality factor and spring constant show, the regions of small eigenfrequency deviation (not perfectly matched) might be the most promising in terms of sensor design as the effective quality factor can be relatively high, i.e., ensure good detectability, while still very good sensitivity is achieved by a low effective spring constant. A more detailed study of the relation between detectability and sensitivity of the co-resonant system based on the derived expressions is necessary in order to fully understand the behaviour of the coupled system in this regard. That will eventually lead to design criteria with respect to different sensor applications.

## Conclusion

Coupling and eigenfrequency matching of a micro- and nanocantilever has experimentally been demonstrated to lead to a significant increase in sensitivity of cantilever sensors while maintaining the ease of detection. This co-resonant measurement principle allows to access the high sensitivity of a nanocantilever as it induces a strong interplay between both individual beams, resulting in an amplitude response curve comprised of two resonance peaks which can be measured at the microcantilever. It was found that these two resonance peaks can be described by effective sensor properties which depend on the individual beam’s properties and the degree of eigenfrequency matching. Consequently, a small eigenfrequency deviation between micro- and nanocantilever results in effective properties for both resonance peaks that are strongly influenced by the nanocantilever’s characteristics. With increasing eigenfrequency deviation, the effective properties of the resonance peaks start to approach those of the individual beams, i.e., one peak is becoming increasingly similar to the single microcantilever while the other one is approaching the nanocantilever’s characteristics. When measuring the coupled system’s amplitude response curve at the microcantilever, this secondly mentioned resonance peak will eventually not be detectable anymore. For sensor design and evaluation of prospective sensor performance it is therefore crucial to analyze and understand the effective sensor properties in order to advance the measurement principle towards sensor implementations for different applications. In this publication, the derivation of analytical expressions for the effective sensor properties resonance frequency, spring constant and quality factor were presented for both resonance peaks of the coupled system based on the coupled harmonic oscillator model. These expressions, in combination with considerations for resonance amplitude amplification between micro- and nanocantilever, allow to easily estimate the prospective sensor performance and gain in sensitivity due to co-resonant coupling based on the parameters of micro- and nanocantilever. This is a crucial contribution for advancing the co-resonant measurement principle and can be the basis for further investigations and applications of co-resonantly coupled systems. Furthermore, it was studied how the thermal noise and consequently the derivation of a minimal detectable frequency shift has to be treated for a co-resonantly coupled system represented by a coupled harmonic oscillator. While calculation steps could be outlined, the solutions are rather complicated due to the complex transfer function of this two-degree of freedom system and will potentially require numerical treatment and elaborate assumptions and approximations. This will be the subject of future investigations and here, a simplified approach to estimate the minimal detectable frequency shift based on the effective sensor properties was shown.

## Supporting Information

File 1 Details on mathematical derivations.

## References

[1] Martínez-Martín D, Fläschner G, Gaub B, Martin S, Newton R, Beerli C, Mercer J, Gerber C, Müller D J (2017). Nature.

[2] Johnson B N, Mutharasan R (2012). Biosens Bioelectron.

[3] Gfeller K Y, Nugaeva N, Hegner M (2005). Biosens Bioelectron.

[4] Baller M K, Lang H P, Fritz J, Gerber C, Gimzewski J K, Drechsler U, Rothuizen H, Despont M, Vettiger P, Battiston F M (2000). Ultramicroscopy.

[5] Stowe T D, Yasumura K, Kenny T W, Botkin D, Wago K, Rugar D (1997). Appl Phys Lett.

[6] Gross B, Weber D P, Rüffer D, Buchter A, Heimbach F, Fontcuberta i Morral A, Grundler D, Poggio M (2016). Phys Rev B.

[7] Gysin U, Rast S, Aste A, Speliotis T, Werle C, Meyer E (2011). Nanotechnology.

[8] Giessibl F J, Pielmeier F, Eguchi T, An T, Hasegawa Y (2011). Phys Rev B.

[9] Melcher J, Stirling J, Cervantes F G, Pratt J R, Shaw G A (2014). Appl Phys Lett.

[10] Li M, Tang H X, Roukes M L (2007). Nat Nanotechnol.

[11] Gil-Santos E, Ramos D, Martínez J, Fernández-Regúlez M, García R, San Paulo Á, Calleja M, Tamayo J (2010). Nat Nanotechnol.

[12] Nichol J M, Hemesath E R, Lauhon L J, Budakian R (2008). Appl Phys Lett.

[13] Reiche C F, Körner J, Büchner B, Mühl T (2015). Nanotechnology.

[14] Körner J, Reiche C F, Büchner B, Mühl T, Gerlach G (2016). J Sens Sens Syst.

[15] Körner J, Reiche C F, Gemming T, Büchner B, Gerlach G, Mühl T (2016). Beilstein J Nanotechnol.

[16] Körner J, Reiche C F, Ghunaim R, Fuge R, Hampel S, Büchner B, Mühl T (2017). Sci Rep.

[17] Reiche C F, Körner J, Büchner B, Mühl T (2015). Bidirectional scanning force microscopy probes with co-resonant sensitivity enhancement. Proceedings of IEEE 15th International Conference on Nanotechnology.

[18] Körner J, Reiche C F, Büchner B, Mühl T (2018). tm - Technisches Messen.

[19] Lochon F, Dufour I, Rebière D (2005). Sens Actuators, B.

[20] Martin Y, Williams C C, Wickramasinghe H K (1987). J Appl Phys.

[21] Jenkins N E, DeFlores L P, Allen J, Ng T N, Garner S R, Kuehn S, Dawlaty J M, Marohn J A (2004). J Vac Sci Technol, B: Microelectron Nanometer Struct–Process, Meas, Phenom.

[22] Gysin U, Rast S, Ruff P, Meyer E, Lee D W, Vettiger P, Gerber C (2004). Phys Rev B.

[23] Ilic B, Yang Y, Craighead H G (2004). Appl Phys Lett.

[24] Rast S, Wattinger C, Gysin U, Meyer E (2000). Rev Sci Instrum.

[25] Voigtländer B (2015). Scanning Probe Microscopy.

[26] Butt H-J, Jaschke M (1995). Nanotechnology.

[27] Weaver W J, Timoshenko S P, Young D H (1990). Vibration Problems in Engineering.

[28] Novotny L (2010). Am J Phys.

[29] Rubbmark J R, Kash M M, Littman M G, Kleppner D (1981). Phys Rev A.

[30] Lang H P, Hegner M, Gerber C, Bhushan B (2007). Nanomechanical cantilever array sensors. Springer Handbook of Nanotechnology.

[R31] 31T. Mühl, personal communication, 24th August 2017.

[32] Pirzer T, Hugel T (2009). Rev Sci Instrum.

[33] Gibson C T, Smith D A, Roberts C J (2005). Nanotechnology.

[34] Morita S, Giessibl F J, Wiesendanger R (2009). Noncontact Atomic Force Microscopy.

[35] Sarid D (1994). Scanning Force Microscopy - With Applications to Electric, Magnetic and Atomic Forces.

[36] Blanter M S, Golovin I S, Neuhäuser H (2007). Internal Friction in Metallic Materials - A Handbook.

[37] Jazar R N (2013). Advanced Vibrations.

[38] Yasumura K Y, Stowe T D, Chow E M, Pfafman T, Kenny T W, Stipe B C, Rugar D (2000). J Microelectromech Syst.

