# Supplementary material for: Effective sensor properties and sensitivity considerations of a dynamic co-resonantly coupled cantilever sensor
Source: Beilstein J Nanotechnol. 2018 Sep 25;9:2546–60. doi: 10.3762/bjnano.9.237 (PMC6176814; doi:10.3762/bjnano.9.237)
Supplement: File 1 — Details on mathematical derivations. [file Beilstein_J_Nanotechnol-09-2546-s001.pdf]

Supporting Information

for

Effective sensor properties and sensitivity considerations of a dynamic co-resonantly coupled cantilever sensor

Julia Körner

Address: University of Utah, 50 S. Central Campus Dr #2110, Salt Lake City, UT-84112, USA

Email: Julia Körner - julia.koerner2k@gmail.com

Details on mathematical derivations

Derivation of frequency shift for dynamic-mode cantilever sensors

The frequency shift $\Delta\omega$ for a dynamic-mode cantilever sensor is generally given as:

$$\Delta\omega = \sqrt{\frac{k+\Delta k}{m_{eff}+\Delta m}} - \sqrt{\frac{k}{m_{eff}}} \quad , \tag{S.1}$$

where k and m_{eff} are the cantilever's spring constant and effective mass, respectively and Δk and Δm denote small changes due to external interactions (force gradient or mass addition).

For negligible mass change, i.e. $\Delta m = 0$, the frequency shift in dependence of a force gradient Δk can be derived by applying the approximation [1]:

$$\sqrt{1+\epsilon} \approx \frac{\epsilon}{2} + 1 \quad , \epsilon << 1 \tag{S.2}$$

for small Δk to the first term in equation (S.1). This results in:

$$\Delta\omega = \sqrt{\frac{k\left(1+\frac{\Delta k}{k}\right)}{m_{eff}}} - \sqrt{\frac{k}{m_{eff}}} \tag{S.3}$$

$$\Delta\omega = \sqrt{\frac{k}{m_{eff}}} \cdot \sqrt{1+\frac{\Delta k}{k}} - \sqrt{\frac{k}{m_{eff}}} \tag{S.4}$$

$$\Delta\omega \approx \sqrt{\frac{k}{m_{eff}}} \cdot \left(\frac{\Delta k}{2k}+1\right) - \sqrt{\frac{k}{m_{eff}}} \tag{S.5}$$

$$\Delta\omega \approx \sqrt{\frac{k}{m_{eff}}} \cdot \frac{\Delta k}{2k} + \sqrt{\frac{k}{m_{eff}}} - \sqrt{\frac{k}{m_{eff}}} \tag{S.6}$$

$$\Delta\omega \approx \omega_0 \cdot \frac{\Delta k}{2k} \quad . \tag{S.7}$$

For negligible Δk, a similar expression can be found for the frequency shift in dependence on the mass change Δm. In this case a Taylor series expansion is employed under the assumption that Δm is small, i.e. at the point $\Delta m = 0$. To do so, equation (S.1) is rewritten:

$$\Delta\omega\left(\Delta m, 0\right) = \sqrt{k} \cdot \left(m_{eff}+\Delta m\right)^{-1/2} - \sqrt{\frac{k}{m_{eff}}} \quad . \tag{S.8}$$

It is sufficient to consider only the first two terms (stationary and first derivative) of the expansion:

$$\Delta\omega\left(\Delta m,0\right)\approx\Delta\omega(0)+\frac{\partial\Delta\omega(0)}{\partial\Delta m}\cdot\left(\Delta m-0\right) \tag{S.9}$$

$$\Delta\omega\left(\Delta m,0\right)\approx 0-\frac{1}{2}\sqrt{k}\left(m_{eff}\right)^{-3/2}\cdot\Delta m \tag{S.10}$$

$$\Delta\omega\left(\Delta m,0\right)\approx -\frac{\Delta m}{2m_{eff}}\cdot\sqrt{\frac{k}{m_{eff}}} \tag{S.11}$$

$$\Delta\omega\left(\Delta m,0\right)\approx -\frac{\Delta m}{2m_{eff}}\cdot\omega_0 \quad . \tag{S.12}$$

Effective properties of the coupled system in dependence on eigenfrequency deviation

Resonance frequencies

The resonance frequencies of the coupled system can be expressed as a function of the eigenfrequency deviation $\Delta\omega_{eigen}=(\omega_2-\omega_1)/\omega_1$:

$$\omega_{a,b}^2=\omega_1^2\cdot\left[\frac{1}{2}\left(K_{12}+K_{23}\cdot\Omega^2\right)\mp\sqrt{\frac{1}{4}\left(K_{12}-K_{23}\cdot\Omega^2\right)^2+\frac{k_2}{k_1}\Omega^2}\right] \quad , \tag{S.13}$$

with

$$\Omega=1+\Delta\omega_{eigen} \quad . \tag{S.14}$$

In that case, the eigenfrequency of the bigger oscillator (1) is assumed to be fixed and only that of the nanocantilever is varied. Please note that this is just a different form of equation (11) from the main text.

Effective spring constants

Based on equation (S.13) and equations (15) and (16) from the main paper, the effective spring constants can also be expressed in dependence on the eigenfrequency deviation, leading to:

$$k_{eff}^{a,b}=\frac{k_3}{2}\frac{\sqrt{\frac{1}{2}\left(K_{12}+\Omega^2\right)\mp\sqrt{\frac{1}{4}\left(K_{12}-\Omega^2\right)^2+\frac{k_2}{k_1}\Omega^2}}}{A} \quad , \tag{S.15}$$

with

$$\begin{aligned}A=&\sqrt{\frac{1}{2}\left(K_{12}+K_{23}\cdot\Omega^2\right)\mp\sqrt{\frac{1}{4}\left(K_{12}-K_{23}\cdot\Omega^2\right)^2+\frac{k_2}{k_1}\Omega^2}}\\&-\sqrt{\frac{1}{2}\left(K_{12}+\Omega^2\right)\mp\sqrt{\frac{1}{4}\left(K_{12}-\Omega^2\right)^2+\frac{k_2}{k_1}\Omega^2}} \quad .\end{aligned} \tag{S.16}$$

References

[1] Bronstein, I. N. and Semendjajew, K. A. and Musiol, G. and Mühlig, H., *Taschenbuch der Mathematik*, Verlag Harri Deutsch, Frankfurt am Main, 2005 (6th edition)
